# Post-Paracentesis Hemoperitoneum From a Bleeding Mesenteric Varix: A Case Report on a Rare Presentation

**DOI:** 10.7759/cureus.21298

**Published:** 2022-01-16

**Authors:** Saad Ali Ansari, Jasninder Singh Dhaliwal, Aditya Desai, Yusra Ansari, Tahir Muhammad Abdullah Khan

**Affiliations:** 1 Internal Medicine, University of California Riverside School of Medicine, Riverside, USA; 2 Internal Medicine, University of Kentucky, Bowling Green, USA; 3 Internal Medicine, Rawalpindi Medical University, Rawalpindi, PAK; 4 Pulmonary and Critical Care Medicine, University of Kentucky, Bowling Green, USA

**Keywords:** portal hypertension, mesenteric varix, hemoperitoneum, paracentesis, cirrhosis

## Abstract

We report a case of a 53-year-old male with decompensated liver cirrhosis secondary to alcohol abuse and hepatitis C infection who was admitted for hemorrhagic shock secondary to upper GI bleed. He underwent a therapeutic paracentesis 17 days after admission with the removal of 6 L of ascitic fluid. The patient became hemodynamically unstable after paracentesis and an acute drop in his hemoglobin was noted. On imaging, he was found to have massive hemoperitoneum secondary to a bleeding mesenteric varix. This is a very rare complication of paracentesis in patients with advanced cirrhosis and should be recognized early in the post-procedure period to initiate prompt life-saving measures to minimize morbidity and mortality.

## Introduction

Hemoperitoneum, defined as blood in peritoneal cavity, is commonly seen because of trauma to the abdomen. Hemoperitoneum secondary to variceal bleeding is rare. Varices in liver cirrhosis are seen because of portal hypertension as increased pressure in portal vein causes backflow and subsequent dilation of portal and anastomosing veins resulting in the formation of varices. Ectopic varices including mesenteric varices are rare causes of bleeding in cirrhotic patients representing only 1-5% of all variceal bleeding [1-2]. In cirrhotic patients, there can be massive amounts of fluid that can be collected in the peritoneal cavity secondary to portal hypertension and hypoalbuminemia which is referred to as ascites. Paracentesis is defined as the act of removing fluid from the peritoneal cavity and it can be diagnostic and therapeutic. Therapeutic paracentesis can be performed to relieve abdominal discomfort and to improve respiratory status in cirrhotic patients on mechanical ventilation [3]. Paracentesis is a relatively safe procedure with a risk of major complications of <2% [4]. Some of the complications can include abdominal wall hematomas, risk of infection, post paracentesis hypotension, risk of bowel perforation, and major bleeding event. We are describing a case of fatal hemorrhagic complication of paracentesis secondary to a bleeding mesenteric varix.

## Case presentation

A 53-year-old male with liver cirrhosis secondary to alcohol abuse and hepatitis C infection with a MELD score of 26, Child Class C presented to the hospital with a one-day history of black tarry stools. He was found to have melena on assessment. Initially, he was alert and oriented in the ED, but he gradually started to become more altered. He was found to be hypoxic and was started on supplemental oxygen. On examination, he had an extremely distended abdomen for which a therapeutic and diagnostic paracentesis was done with removal of 5 L of straw-colored ascitic fluid. Fluid analysis was not suggestive of spontaneous bacterial peritonitis. He was noted to have a hemoglobin of 6.2 g/dL (12.5-16.3), and he received 2 units of packed red blood cell (PRBC), and he was started on octreotide drip and pantoprazole. He was hemodynamically unstable with blood pressure (BP) of 60/40 mm Hg and was started on norepinephrine support. He underwent an emergent esophagogastroduodenoscopy (EGD) with banding done on three bleeding esophageal varices. Due to his worsening encephalopathy and respiratory distress and inability to protect his airway he had to be intubated and put on ventilatory support.

He had another therapeutic paracentesis done on his 7th day of admission with the removal of 1.2 L of ascitic fluid without complications. Due to worsening renal function, he was started on hemodialysis on his 10th day of admission. Therapeutic paracentesis was repeated on the 13th day of admission with the removal of 2 L of ascitic fluid. Over the next day, the patient started to have increasing vasopressor requirement. His hemoglobin dropped from 8.5 g/dL to 5.1 g/dL and coffee ground discharge was noted via nasogastric tube. He was started on massive transfusion protocol and GI repeated EGD with further banding of three bleeding esophageal varices. Over the next two days, the patient became more stable with improving vasopressor and ventilator requirements.

**Figure 1:**
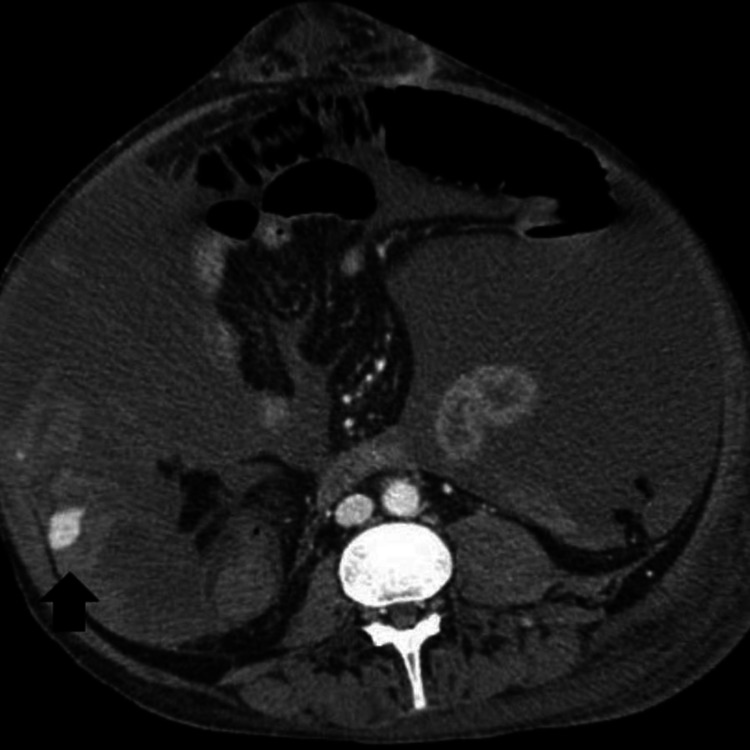
CT with angiography showing hemoperitoneum with hemorrhagic mesenteric varix (black arrow); axial view.

**Figure 2:**
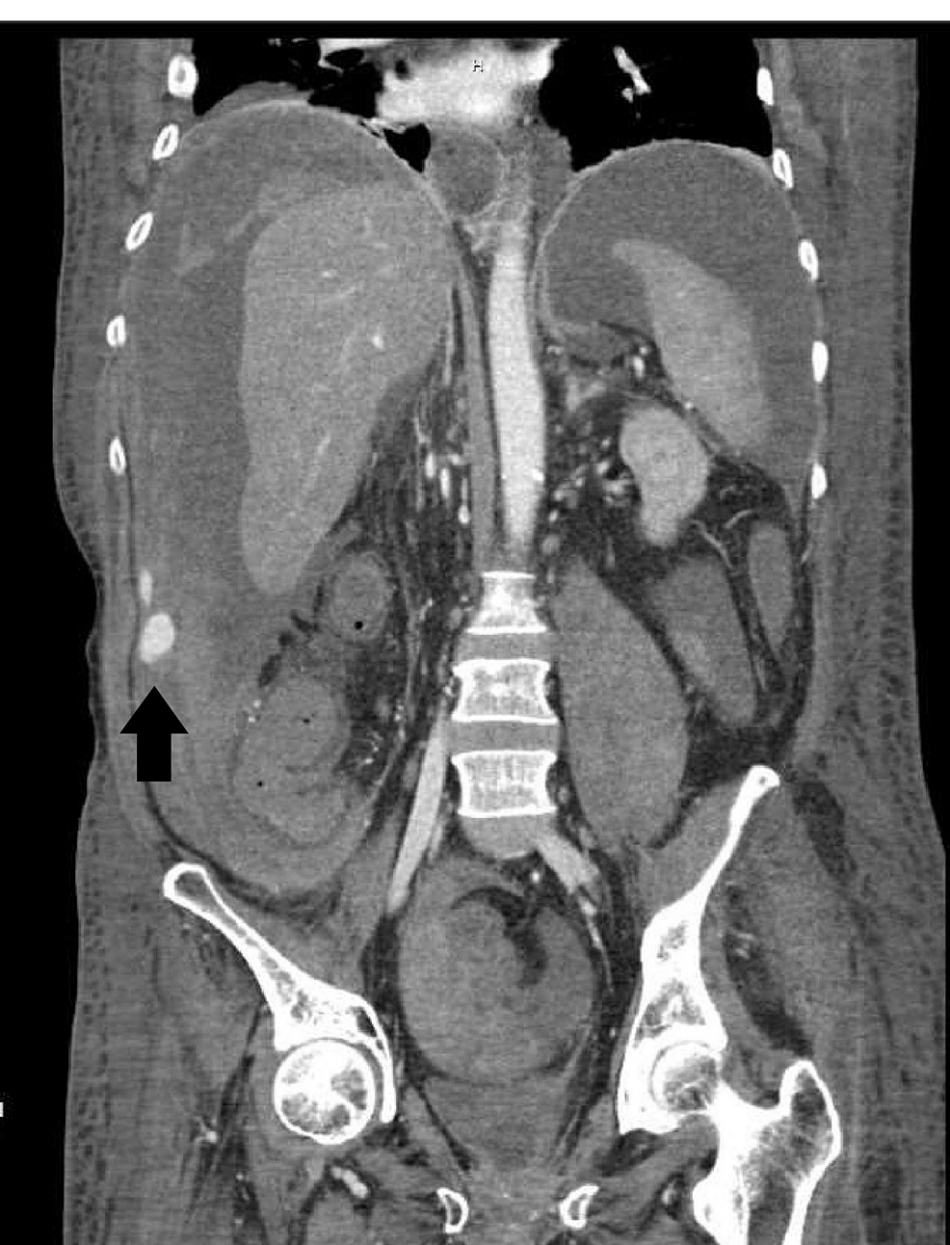
CT with angiography showing hemoperitoneum with hemorrhagic varix (black arrow); coronal view.

**Figure 3:**
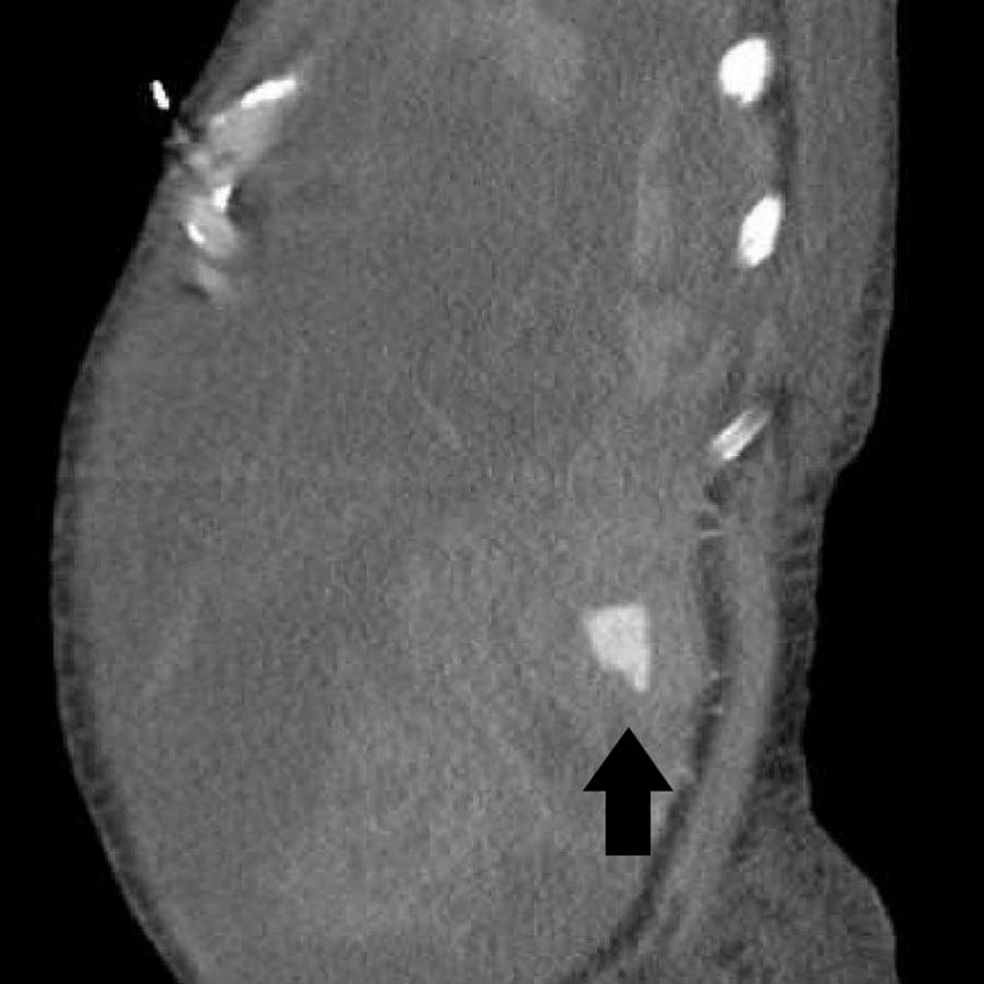
CT with angiography showing hemoperitoneum with hemorrhagic mesenteric varix (black arrow); sagittal view.

On the 17th day of admission, he was noted to have increased abdominal distention with point-of-care ultrasound (POCUS) showing a good amount of ascitic fluid. Further therapeutic paracentesis was planned. Before the procedure, his international normalized ratio (INR) was noted to be 3.0 (0.8-1.2) and he had a platelet count of 23 K/uL (130-400 K). A good fluid pocket was identified on ultrasound and a paracentesis was performed with a right lateral approach. A total of 6 L of straw-colored ascitic fluid was removed. The patient was given albumin after the procedure to avoid post-paracentesis hypotension. Overnight the patient became hemodynamically unstable with the increasing vasopressor requirement. His hemoglobin was noted to have dropped to 3.8 g/dL from 8.7 g/dL. Massive transfusion protocol was initiated. CT angiography abdomen and pelvis was done which showed massive hemoperitoneum. An active bleeding varix in the right lower quadrant of the abdomen arising from superior mesenteric vein vasculature was identified on portal venous phase imaging of CT (Figures 1-3).

The patient was also found to be in suspected disseminated intravascular coagulation with a significant amount of oral bleeding noted. The patient's family opted against any further intervention and they decided to put the patient on comfort measures. The patient passed away the following day.

## Discussion

Hemoperitoneum is very rarely seen secondary to variceal bleeding. Abdominal varices are dilated veins with a high risk of bleeding and are commonly seen in patients with cirrhosis as a consequence of portal hypertension. Ectopic varices comprise large portosystemic venous collaterals located anywhere other than the gastro-esophageal region and they are responsible for only 1-5% of all variceal bleeding [1-2].

Although variceal bleeding is a common cause of mortality in patients with liver cirrhosis, severe or fatal hemorrhagic event following large-volume paracentesis is extremely rare [4-5]. Bleeding from mesenteric varix is even rarer and is responsible for around 20% of all hemorrhagic events following paracentesis, however, it is the most common cause of hemoperitoneum following paracentesis [6]. In general, patients with liver cirrhosis and abnormal coagulation studies with thrombocytopenia do not require any fresh frozen plasma or platelet transfusion before paracentesis as deranged coagulation profile is not an accurate predictor of bleeding risk in cirrhotic patients, however, a low fibrinogen can sometimes be an independent predictor of bleeding risk [7-10].

Even though the risk is extremely low, it is important to be aware of hemoperitoneum and hemorrhage as a potential consequence of abdominal paracentesis. Every step should be taken to minimize bleeding risk including using ultrasound to identify a good fluid pocket and to mark the site for needle insertion. Tests like fibrinogen level and thromboelastography can potentially help in identifying patients at higher risk of bleeding, but these tests may not always be possible or available.

Early identification of a bleeding episode is extremely important. Bleeding should always be suspected after paracentesis if the patient becomes more hemodynamically unstable or develops increasing abdominal distention or if there is an acute drop in hemoglobin. Immediate efforts to stabilize the patient and to achieve hemostasis should be initiated. Massive blood transfusion protocol should be ordered and vasoactive agents like octreotide and terlipressin should be started to reduce splanchnic blood flow, and Interventional Radiology should be involved in patient care for immediate intervention. Potential options include catheter-directed embolization and balloon-occluded retrograde transvenous obliteration either alone or in combination with transjugular intrahepatic portosystemic shunt (TIPS). If this is not successful, the patient may have to be taken to the OR for exploratory laparotomy [2,11].

## Conclusions

In conclusion, ultrasound-guided abdominal paracentesis is an extremely safe procedure with very few complications. Hemoperitoneum is an extremely rare complication of paracentesis. We presented a case of fatal hemoperitoneum secondary to bleeding ectopic mesenteric varix following large-volume paracentesis. Immediate recognition of this complication in the clinical setting is important in order to minimize mortality.
